# Prevalence and Impact of Atrial Fibrillation in Hospitalized Patients with COVID-19: A Systematic Review and Meta-Analysis

**DOI:** 10.3390/jcm10112490

**Published:** 2021-06-04

**Authors:** Giulio Francesco Romiti, Bernadette Corica, Gregory Y. H. Lip, Marco Proietti

**Affiliations:** 1 Department of Translational and Precision Medicine, Sapienza—University of Rome, 00161 Rome, Italy; giuliofrancesco.romiti@uniroma1.it (G.F.R.); bernadette.corica@uniroma1.it (B.C.); 2 Liverpool Centre for Cardiovascular Science, University of Liverpool and Liverpool Heart & Chest Hospital, Liverpool 14 3PE, UK; gregory.lip@liverpool.ac.uk; 3 Department of Clinical Medicine, Aalborg University, DK-9100 Aalborg, Denmark; 4 Geriatric Unit, IRCCS Istituti Clinici Scientifici Maugeri, 20138 Milan, Italy; 5 Department of Clinical Sciences and Community Health, University of Milan, 20122 Milan, Italy

**Keywords:** atrial fibrillation, COVID-19, prognosis, outcomes

## Abstract

Background: In patients with COVID-19, cardiovascular complications are common and associated with poor prognosis. Among these, an association between atrial fibrillation (AF) and COVID-19 has been described; however, the extent of this relationship is unclear. The aim of this study is to investigate the epidemiology of AF in COVID-19 patients and its impact on all-cause mortality. Methods: A systematic review and meta-analysis were performed and reported according to PRISMA guidelines, and a protocol for this study was registered on PROSPERO (CRD42021227950). PubMed and EMBASE were systematically searched for relevant studies. A random-effects model was used to estimate pooled odds ratios (OR) and 95% confidence intervals (CI). Results: Overall, 31 studies were included in the analysis, with a total number of 187,716 COVID-19 patients. The prevalence of AF was found to be as high as 8% of patients with COVID-19 (95% CI: 6.3–10.2%, 95% prediction intervals (PI): 2.0–27.1%), with a high degree of heterogeneity between studies; a multiple meta-regression model including geographical location, age, hypertension, and diabetes showed that these factors accounted for more than a third of the heterogeneity. AF COVID-19 patients were less likely to be female but more likely older, hypertensive, and with a critical status than those without AF. Patients with AF showed a significant increase in the risk of all-cause mortality (OR: 3.97, 95% CI: 2.76–5.71), with a high degree of heterogeneity. A sensitivity analysis focusing on new-onset AF showed the consistency of these results. Conclusions: Among COVID-19 patients, AF is found in 8% of patients. AF COVID-19 patients are older, more hypertensive, and more likely to have a critical status. In COVID-19 patients, AF is associated with a 4-fold higher risk of death. Further studies are needed to define the best treatment strategies to improve the prognosis of AF COVID-19 patients.

## 1. Introduction

From early 2020, COVID-19 has caused a high burden of morbidity and mortality worldwide [1]. Cardiovascular diseases were indicated among the leading causes of clinical deterioration and adverse outcomes in these patients [2], and the association between COVID-19 and cardiac arrhythmia has been described [3].

Atrial fibrillation (AF) is among the most common cardiac arrhythmias, and its association between infectious diseases and critical illness has already been reported [4,5], with a significant burden on management and prognosis [6,7]. Several hypotheses have been proposed to explain the relationship between AF and infection, including cytokine storm, inflammation, and oxidative stress [5,8]. All these phenomena are also commonly reported in COVID-19 patients [9].

Notwithstanding this, only few studies have reported about the onset of AF during COVID-19 [3,10], and the actual prevalence and the impact of AF on the prognosis of these patients are still unclear. On the other hand, AF is already recognized as the most common arrhythmia occurring during COVID-19 [3,11,12]. This emphasizes the potential for a tight relationship between these two diseases, as well as the clinical relevance of this association. Although previous systematic reviews and meta-analyses have investigated the relationship between AF and COVID-19, these were based on a limited number of patients, were not focused on the AF episodes occurring during COVID-19, or did not provide an extensive study of heterogeneity between studies [13,14,15]. A more comprehensive evaluation of the relationship between AF and COVID-19, also taking into account those factors that may influence the prevalence of AF and its related outcomes, is highly warranted to inform physicians involved in the frontline of this pandemic [16].

We aimed to perform a systematic review and meta-analysis of the prevalence of AF, factors associated with its onset, and the impact on all-cause mortality in patients with COVID-19.

## 2. Methods

This systematic review was performed according to the Meta-analysis Of Observational Studies in Epidemiology (MOOSE) guidelines and reported according to the Preferred Reporting Items for Systematic Reviews and Meta-Analyses (PRISMA) guidelines. A protocol for this study was registered into the international prospective register of systematic reviews (PROSPERO), N. CRD42021227950.

### 2.1. Search Strategy

A systematic literature search was performed on PubMed and EMBASE databases, from inception to 10 March 2021. Relevant key terms were combined in the search strategy, including ‘Sars-CoV-2′, ‘COVID-19′, and ‘atrial fibrillation’. The full search strategy is reported in Supplementary Materials (Supplementary Table S1).

### 2.2. Studies Selection

All articles retrieved from the literature search were systematically screened independently by two investigators (G.F.R. and B.C.) according to titles and abstracts. Each article included after the first screening phase was then evaluated independently by two investigators (G.F.R. and B.C.) according to full-text eligibility. Disagreements were resolved by collegial discussion with a third author (M.P.).

### 2.3. Inclusion and Exclusion Criteria

The main inclusion criteria were: (i) all studies reporting the prevalence of AF in hospitalized patients with COVID-19; (ii) all studies reporting outcomes (i.e., mortality) according to AF status in patients with COVID-19. For the purpose of this study, AF was defined as the occurrence of AF during COVID-19, as defined in the original studies, and irrespective of the previous history of AF. When a study reported on AF and atrial flutter together, we included all patients under the AF definition, unless a clear distinction was possible based on published data. We excluded studies on highly selected cohorts (e.g., only deceased patients) of patients with COVID-19, conference abstracts, comments, editorials, case reports, systematic reviews, and meta-analyses. In the case of duplicated cohorts (i.e., two or more studies based on the same cohort of patients), we selected the study with (i) the highest number of patients included, (ii) the most complete set of information, or (iii) the most recently published one.

### 2.4. Data Extraction and Quality Assessment

Data from the studies included were independently extracted by two investigators (G.F.R. and B.C.) with the use of a standardized electronic form. We extracted data on sample size, numbers of patients with AF, proportion of females, and prevalence of several comorbidities (including hypertension, diabetes mellitus, coronary artery disease (CAD), chronic heart failure (CHF), and chronic kidney disease (CKD)). We also extracted data on the proportion of “critical” patients; since studies heterogeneously reported characteristics related to COVID-19 severity, we defined “critical” patients as follows: (i) patients were admitted to the ICU, (ii) patients underwent mechanical ventilation, or (iii) patients were defined as “critical” in the original studies. Additionally, we extracted data regarding outcomes (in-hospital death or 30-day mortality) according to the AF status, when available.

All studies included were independently evaluated by two investigators (G.F.R. and B.C.) to assess the risk of bias. According to the outcomes investigated, we evaluated the risk of bias separately for each outcome of the study: for the prevalence of AF, we assessed the risk of bias using a customized version of the Newcastle–Ottawa scale (NOS) for cross-sectional studies, composed of five items across three domains (selection, comparability, outcome), with a maximum of 5 points. Any study with a score ≤3 was categorized as being at a high risk of bias. For studies exploring outcomes according to AF status, we assessed the risk of bias using a customized version of the NOS for cohort studies [17], composed of eight items across three domains (selection, comparability, outcome). Any study with a score ≤6 was categorized as being at a high risk of bias.

### 2.5. Outcomes Definition

Prevalence of AF was defined as the proportion of patients that present AF at admission or during the clinical course of COVID-19, as defined in the original studies.

We also investigated the association of AF with several conditions or baseline characteristics of the COVID-19 patients, including age, female sex, history of hypertension, diabetes mellitus, coronary artery disease (CAD), and chronic heart failure (CHF), as well as the critical disease status as previously defined.

All-cause mortality was defined as the occurrence of in-hospital death or 30-days death in patients with COVID-19, according to AF status.

### 2.6. Statistical Analysis

The prevalence of AF reported in the original studies included were pooled with a random intercept logistic regression model [18], and reported as pooled prevalence, 95% confidence intervals (CI), and 95% prediction intervals (PI). PI represents a predicted range of the true effect in a potential future study, and provides useful information on the variability of the effect in different clinical settings [19,20].

Dichotomous variables were pooled and compared using random-effects models, and reported as odds ratios (OR) mean difference and 95% CI. For continuous variables, mean, SD, and total number in each group were pooled and compared with the inverse variance method; mean difference and 95% CI were reported accordingly.

The inconsistency index (I^2^) was calculated to measure heterogeneity. According to pre-specified cut-offs, low heterogeneity was defined as an I^2^ of <25%, moderate heterogeneity when I^2^ fell between 25 and 75%, and high heterogeneity when I^2^ was >75%.

For prevalence of AF and all-cause mortality, a sensitivity analysis was performed with a “leave-one-out” approach, in which all studies are removed iteratively one at a time to evaluate their influence on the pooled estimate and heterogeneity. As an additional sensitivity analysis, we also computed the prevalence of AF according to the inverse variance method, with two types of transformation of the proportions (logit transformation and Freeman–Tukey double arcsine transformation).

To investigate the potential sources of heterogeneity in the pooled prevalence of AF, we performed several subgroup analyses, according to geographical location, study design (retrospective vs. observational), and risk of bias. Moreover, we also performed univariable and multivariable meta-regression, according to study-level relevant baseline characteristics, to identify potential study-level characteristics associated with the prevalence of AF, or the risk of all-cause mortality in patients with AF vs. those without AF.

A sensitivity analysis, including only patients with new onset AF (i.e., patients who presented with AF during COVID-19, with the exclusion of those with a previous history of AF) was also performed for both the prevalence of AF and all-cause mortality. For this analysis, we excluded from the calculation those patients with a known history of AF. Only those studies for which these data were available (or derivable from the main cohort), and clearly referred to new onset AF, were included in this analysis.

Publication bias was assessed for studies reporting all-cause mortality according to AF status, with the use of funnel plots, which were visually inspected for asymmetricity. Egger’s test was also performed. In case of detection of significant publication bias, we performed further analysis according to the trim-and-fill approach [21], in which additional studies which correct for the asymmetry in the funnel plot are imputed and combined with those included in the meta-analysis to analyze the actual effect of potential publication bias.

All the statistical analyses were performed using R version 4.0.3 (R Core Team, R Foundation for Statistical Computing, Vienna, Austria, 2020), with the use of ‘meta’ [22], ‘metafor’ [23], and ‘dmetar’ [24] packages.

## 3. Results

Of the 783 studies identified from the literature search (216 on Pubmed and 567 on EMBASE), 31 studies were selected according to the inclusion and exclusion criteria and included in the analysis [3,10,11,12,25,26,27,28,29,30,31,32,33,34,35,36,37,38,39,40,41,42,43,44,45,46,47,48,49,50,51] (Figure S1 in Supplementary Materials), with a total of 187,716 COVID-19 patients. Baseline characteristics of the studies included are reported in Table 1.

Thirteen studies were held in Europe [10,11,25,29,31,33,37,39,42,43,44,47,50], nine in North America [3,28,30,32,41,45,46,49,51], four in Asia [26,34,35,48], and five in other geographical locations, including one multinational study [12,27,36,38,40]. Twenty-five studies were observational retrospective [3,10,12,26,28,30,31,32,34,35,37,38,39,40,41,42,43,44,45,46,47,48,49,50,51], four were based on prospective observational analysis of single center cohorts [11,25,29,36], while two were observational prospective multicenter studies [27,33].

Of the studies, 14 were included in the analysis for all-cause mortality according to AF status (12 reported in-hospital death [3,10,12,25,29,30,37,40,41,42,43,45] and two described 30-day mortality [32,50]). Nine studies included patients with new onset AF [10,29,36,37,40,41,42,45,51]. To improve the reliability of our estimates, after careful evaluation of the definitions of AF used and of the data reported, for two studies [37,51], we decided to compute only new onset AF in the main analysis about prevalence.

The evaluation of bias for the two outcomes investigated is reported in Supplementary Materials (Tables S2 and S3, respectively). Among studies reporting the prevalence of AF, 13 studies were categorized as being at high risk of bias [11,32,33,34,36,37,39,42,44,46,47,48,51]; incomplete reporting of baseline characteristics was the most common concern. Among the studies reporting all-cause mortality according to AF, two studies were at high risk of bias [42,50].

### 3.1. Prevalence of AF in Patients with COVID-19

**Figure 1:**
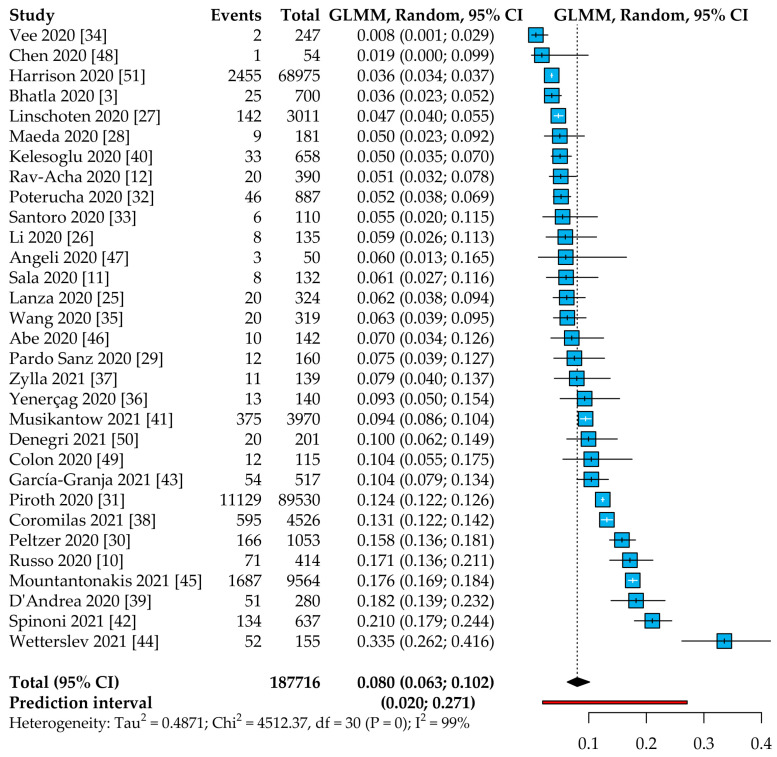
Pooled prevalence of AF in COVID-19 patients. Legend: CI = confidence interval; GLMM = general linear mixed model.

In the 31 studies included, 8.0% of patients with COVID-19 presented AF (95% CI: 6.3–10.2%, 95% PI: 2.0–27.1%), with high heterogeneity between studies (Figure 1). The leave-one-out sensitivity analysis showed consistent results (Supplementary Figure S2); similar estimates were also found in the sensitivity analysis according to the inverse-variance method, using different methods for transformation of the proportions (Supplementary Table S4).

Pre-specified subgroup analyses are reported in Supplementary Table S5. Significant differences were observed according to the geographical location of the included studies, with the European-based cohorts showing a higher prevalence of AF (11.3%) compared to other studies performed in North America or Asia/other geographical locations (7.5% and 5.3%, respectively). No significant differences were observed according to the study design or the risk of bias.

**Figure 2:**
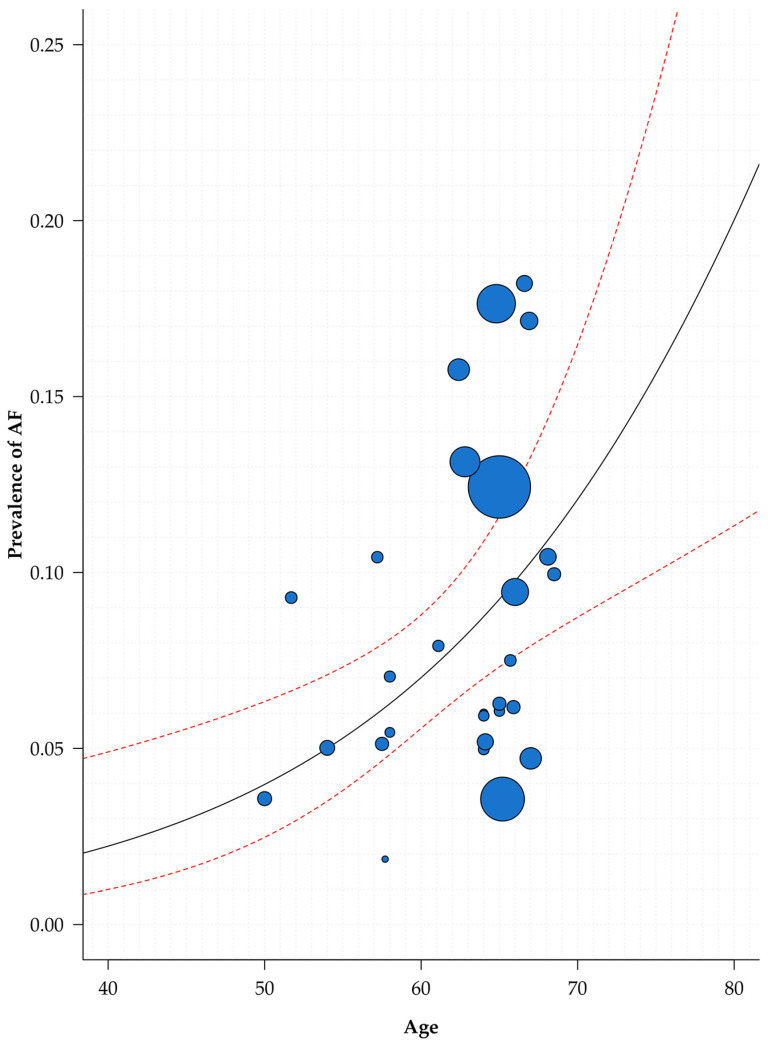
Relationship between age and AF prevalence in COVID-19 patients. Legend: AF = atrial fibrillation.

The results of the meta-regressions are reported in Table 2. At univariable analysis, only age and hypertension resulted significantly and directly associated to the prevalence of AF; a similar, non-significant trend was also observed for geographical location and diabetes (Table 2). A multivariable meta-regression model comprising geographical location, mean age, history of hypertension, and history of diabetes was able to explain part of the heterogeneity observed (R^2^ = 46.0%, *p* = 0.019). A graphical representation of the relationship between the mean age of studies included and the prevalence of AF is reported in Figure 2.

### 3.2. Clinical Characteristics of AF Patients with COVID-19

We compared several baseline characteristics and conditions between patients presenting with and without AF. Eight studies reported data disaggregated by AF diagnosis on proportion of female sex, hypertension, and diabetes [10,29,30,37,40,41,43,45]; seven reported about mean age [10,29,30,37,40,43,45] and CHF [10,29,30,40,41,43,45], while six reported on CAD [10,29,30,41,43,45]; finally, 12 reported on proportion of critical patients [3,26,29,30,35,37,40,41,45,48,49] (specifically, six according to ICU admission [3,26,29,30,43,49], two according to “critically ill” definition [35,48], one according to ICU/intermediate care unit admission [37], and two according to patients receiving mechanical ventilation [41,45]).

Results for this analysis are reported in Table 3. AF was associated with older age (mean difference: 13.2 years, 95% CI 10.5–15.9) and a 17% lower likelihood of being female. AF patients were more likely affected by hypertension, diabetes mellitus, CAD, or CHF; finally, they had a 3.6-fold higher chance of having a critical COVID-19 clinical course. Low to moderate heterogeneity was found for all analyses, except for mean age, which showed high heterogeneity (I^2^ = 86%).

### 3.3. All-Cause Mortality according to AF Status

**Figure 3:**
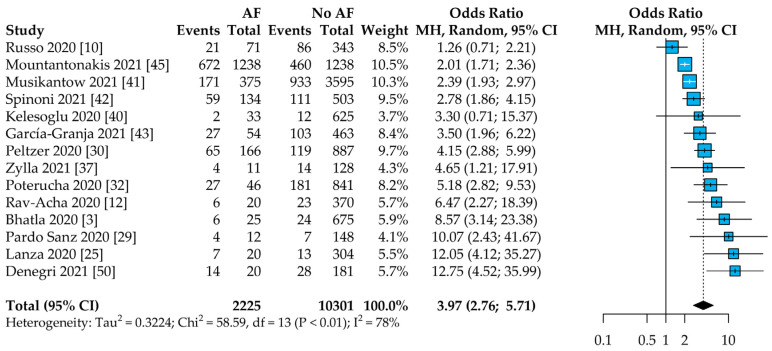
Risk of all-cause mortality in AF vs. non-AF patients. Legend: AF = atrial fibrillation; CI = confidence interval; MH = Mantel–Haenszel.

In the 14 studies reporting on all-cause mortality among COVID-19 patients and according to AF status, patients with AF showed a 4-fold higher risk of death compared to patients without AF (OR: 3.97, 95%CI: 2.76–5.71, Figure 3), with high heterogeneity found between studies (I^2^ = 78%). The leave-one-out analysis showed little to no effect of single studies on the results (Figure S3).

Subgroup analyses according to the outcome definition are reported in Figure S4. Twelve studies reported in-hospital death [3,10,12,25,29,30,37,40,41,42,43,45] and two reported 30-days mortality [32,50]. Patients with AF showed both higher risk of in-hospital mortality (OR 3.52, 95% CI 2.44–5.10) and 30-days mortality (OR 7.34, 95% CI: 3.11–17.34), with no statistically significant difference between subgroups (*p* = 0.12; Figure S4).

Finally, we performed univariable and multivariable meta-regression analyses for all-cause mortality. None of the predictors examined were found to be significantly associated with risk of all-cause death (Table S6).

Significant publication bias was found across the 13 studies included in the analysis (Egger’s test *p* = 0.002, Figure S5). Visual inspection of the funnel plot revealed a void in the lower-left part of the diagram. The correction of the asymmetry of the funnel plot according to the trim-and-fill approach did not affect the significance of the results, despite lowering the pooled estimate (OR: 2.49, 95% CI: 1.58–3.92), suggesting that publication bias is unlikely to contribute to the overall significance of our results.

### 3.4. Sensitivity Analysis about New-Onset AF

In this pre-specified sensitivity analysis about COVID-19 patients with a new-onset AF (thus with the exclusion of those with a diagnosis of AF before occurrence of COVID-19), nine studies were included for the prevalence of AF [10,29,36,37,40,41,45,51] and five for all-cause mortality according to the AF status [10,37,40,42,45]. The prevalence of new-onset AF was found as high as 7.4% (95% CI: 5.3–10.2%, Figure S6 Panel A in Supplementary Materials), with a high grade of heterogeneity found between studies. New-onset AF was also associated with a significant 2.4-fold higher risk of all-cause mortality compared to patients with no AF (Figure S6, Panel B), with a low degree of heterogeneity found between studies.

## 4. Discussion

In this systematic review and meta-analysis of 187,716 adults, AF was found in 8% of COVID-19 patients; high heterogeneity was found between studies, and the 95% PI indicated that actual prevalence of AF maybe up to 27%. AF COVID-19 patients were more likely to be older, hypertensive, diabetic, with concomitant CAD and CHF, and in a critical clinical status. Third, the presence of AF in COVID-19 patients was associated with a 4-fold higher risk of death. Overall, there was consistency in all the sensitivity analyses performed, even if confined to patients with new-onset AF.

Previous meta-analyses tried to investigate the relationship between AF and COVID-19. However, these were based on significantly fewer studies and patients, and provided no information on the potential reasons of the heterogeneity found [13,15]; similarly, another systematic review that focused on outcomes [14] was not specifically designed to assess the prognostic role of AF developing during COVID-19, since it also included those studies that reported outcomes according to a pre-existing history of AF.

Compared to these previous reports, our study has several strengths. First, we provided a comprehensive and updated search and included more studies and a higher number of patients, encompassing both the epidemiology of AF and the related risk of death in patients with COVID-19; second, we focused only on those AF episodes that developed during COVID-19, so that the relationship observed may be more clinically relevant compared to that between history of AF and COVID-19; third, we calculated and reported 95% PI for our estimates of AF prevalence, providing information on the variability of the prevalence in different clinical settings [19,20]; finally, we performed an extensive assessment of the heterogeneity observed between studies, identifying potential moderators of the association between COVID-19 and AF.

Our findings make it possible to postulate on the pathophysiological mechanisms underlying the association between AF and COVID-19. The relationship between infections, inflammation, and the onset of AF is well known, although not completely explained [5]. Cytokine storm, oxidative stress, and atrial remodeling are among the putative phenomena that may trigger the onset of AF, especially in patients with an individual predisposition [5]; moreover, inflammation has been linked to complex cellular changes in atrial myocardiocytes, which can ultimately contribute to the development of an arrhythmogenic milieu [52]. COVID-19 has been described from inception as an inflammatory disease [53], and targeting the dysregulated immune response was postulated as a promising therapeutic approach [54,55,56,57,58]. Indeed, the relationship between AF and COVID-19 may be—at least partially—explained by the increased burden of systemic inflammation. For example, higher levels of C-reactive protein and interleukin-6 were observed in COVID-19 patients with AF, compared to patients without AF [37].

Inflammation may also trigger arrhythmias through determining a more severe clinical course of COVID-19. Inflammatory burden during COVID-19 was associated with greater disease severity [59] and increased risk of death [60]. On the other hand, the association between critically ill status and the onset of AF is well established, particularly during infections [7]; indeed, AF in our study was associated with a five-fold greater chance of having a critical clinical state.

Taken together, these findings may be interpreted as the results of a complex interplay between systemic inflammation, clinical status, and the onset of AF. Consistently, inflammation has been indicated as one key risk factor that may trigger AF onset during critical illness [61], and these multiple interacting mechanisms may also be involved in patients with COVID-19.

The prevalence of AF that we found is consistent with other studies focused on non-COVID-19 pneumonia, which reported similar or slightly higher prevalence of AF [62,63]. Although a higher prevalence of AF may have been expected in COVID-19 patients, two remarks should be made. First, the mean age and the burden of comorbidities found in most studies were relatively low, compared to usual general population AF cohorts, thus indicating an overall low individual risk of AF. Second, significant heterogeneity was found between studies, with the 95% PI pointing towards a potentially higher prevalence of AF in further studies. Due to inconsistent reporting in the (early) original studies, only few factors were available for our meta-regression analysis, which showed that the combination of age, hypertension, diabetes mellitus, and geographical location may explain more than 40% of the heterogeneity observed. Even amongst the subset of studies investigating new-onset AF, the results indicate that the burden of risk factors may have a major role in the development of arrhythmia. While the strict relationship between increasing age and AF is largely known [64], as well as the association between older age and increased mortality during COVID-19 [65], our data help underline how older age may represent a “proxy” for clinical complexity, providing a substrate for developing conditions strongly connected with clinical complexity and multimorbidity, such as AF [66]. Consistently, a recent systematic review showed that increasing age, male sex, and cardiovascular comorbidities were strong risk factors for the onset of AF in ICU patients [61]; the association between these conditions, as well as multimorbidity, with disease severity and worse prognosis in patients with COVID-19 has also already been highlighted [67,68]. These findings suggest that AF may represent a “marker” of additional risk in COVID-19 patients. Indeed, patients with AF have a 4-fold increased risk of all-cause death.

The link between AF and mortality in COVID-19 patients could be explained by several mechanisms. Beyond the relationship with inflammation and multimorbidity, AF may directly worsen prognosis through hemodynamic instability, thromboembolism [69], and increased endothelial dysfunction [70]. Additionally, the results of the meta-regression analysis, which did not identify any moderators of the risk of mortality, strengthen the idea that AF may have a direct effect in increasing mortality in COVID-19 patients. This hypothesis is also supported by our sensitivity analysis on patients with new-onset AF, which showed a consistent 2.8-fold high risk of all-cause death, and by the disaggregated analysis of in-hospital mortality and 30-days mortality, which gave broadly comparable results. The phenotype of AF, including duration and recurrent episodes, may also impose different prognosis in COVID-19 patients; however, due to insufficient data reported in the original studies, we were unable to explore the contribution of specific AF characteristics on the risk of all-cause mortality in these patients. Moreover, uncertainties exist on the risk of AF recurrence in the long-term follow-up of patients with COVID-19. One large study found that over one third of patients with infection-related AF presented arrhythmia recurrence in the first year of follow-up [4], but whether this also applies to the COVID-19 scenario needs to be identified and confirmed, as well as the potential predictors of AF recurrence.

Finally, our results leave questions open on the appropriate management of patients who develop AF during COVID-19, particularly regarding thromboembolic risk and the implementation of antithrombotic treatment. The role of specific therapies, including immune modulators, antiarrhythmics, and antithrombotics on the prognosis of AF and COVID-19 patients is still far from being elucidated, and further studies are needed to understand whether specific strategies may have an influence on the outcome of these subjects. Although no definitive guidance exists on the management of new-onset AF occurring during infections, a recent large study found that these patients may experience a high thromboembolic risk [4], suggesting that they may need to be managed similarly to patients with non-infection related AF, also consistent with the pathophysiological hypotheses reported above. Moreover, the choice of antiarrhythmics for patients who develop AF during infections is still an open debate, although some potentially relevant prognostic differences were outlined recently [71,72]. Ultimately, the role of immunomodulating therapies in this clinical scenario is still unclear and needs further specific investigation [73]. From a broader perspective, as AF occurring during COVID-19 may indicate increased clinical complexity, these patients should be carefully evaluated for the presence of other clinical conditions. The use of an integrated approach which aims, among other things, at the optimal management of comorbidities, is currently endorsed by the recent European AF guidelines [64], and was found to be effective in improving prognosis [74]. It is plausible that a similar approach would also be beneficial in the clinical settings of patients who experience AF during COVID-19, although further studies are needed to define the prognosis and the optimal management of these subjects.

### Limitations

Our study has several limitations. First, most studies were retrospective or not specifically designed to estimate the prevalence of AF or its effect on outcomes. However, this is a common limitation of many epidemiologic studies investigating the relationship between comorbidities and COVID-19; moreover, we provided 95% PI values, which are useful to quantify the amount of uncertainty in our estimates, and performed meta-regressions to investigate potential sources of heterogeneity. Second, the definition of “critical clinical disease” that we used may be prone to bias. However, the association between AF and critical status was strong, with moderate heterogeneity between studies. Third, a clear distinction between patients with new-onset AF and patients with both AF and previous history of arrhythmias was available only in a subset of studies. Nonetheless, the sensitivity analysis based on new-onset AF showed consistent results compared to the main analysis. Fourth, according to the data availability from the original studies, we were unable to assess the impact of other potentially relevant study-level characteristics (including other comorbidities and previous medical conditions, smoking habit, alcohol use, etc.) on the epidemiology of AF during COVID-19, as well as their impact on prognosis. Finally, we were unable to explore inflammatory burden differences between AF and non-AF patients, as well as the impact of different types and duration of AF episodes, or the contribution of a specific treatment, on the prognosis of COVID-19 patients, since most studies did not report laboratory parameters according to the AF status, information on AF phenotype, or data on the prognosis according to treatment received.

## 5. Conclusions

In this systematic review and meta-analysis, the prevalence of AF was 8% patients with COVID-19, and was associated with older age, male sex, hypertension, and critical status. Patients with AF showed a 4-fold higher risk of all-cause mortality compared to those without AF. Similar findings were observed in patients with new-onset AF. Our analysis underlines the detrimental role of AF in patients with COVID-19 and supports the need for the implementation of specific and tailored strategies for the prevention, diagnosis, and management of patients with COVID-19 and concurrent AF. Further studies are needed to define the optimal strategies for the follow-up of these patients, particularly regarding antithrombotic strategies.

## Figures and Tables

**Table 1:** Main characteristics of the studies included in the systematic review.

Study	Geographic Location	Study Type	Incl./Excl. Criteria	AF Diagnosis	N	AF	Age (Mean)	F (%)	HTN (%)	DM (%)	CRIT (%)	Previous AF (%)	Outcome
Abe 2020 [46]	United States	Retrospective	Hospitalized patients	ECG changes	142	9	58	50	73	50	NA	11	NA
Angeli 2020 [47]	Italy	Retrospective	Hospitalized patients	Baseline ECG	50	3	64	28	50	12	0	NA	NA
Bhatla 2020 [3]	United States	Retrospective	Hospitalized patients	ECG changes	700	25	50	55	50	26	11	6	In-hospital mortality
Chen 2020 [48]	China	Retrospective	Severe hospitalized patients	ECG changes	54	1	57.7	33	30	46	100	NA	NA
Colon 2020 [49]	United States	Retrospective	Hospitalized patients	ECG, telemetry	115	12	57.2	54	70	39	60	5	NA
Coromilas 2021 [38]	Multinational	Retrospective	Hospitalized patients	Baseline ECG	4526	595	62.8	43	55	35	20	9	NA
D’Andrea 2020 [39]	Italy	Retrospective	Hospitalized patients	ECG	280	51	66.6	40	35	NA	NA	NA	NA
Denegri 2021 [50]	Italy	Retrospective	ED admission	Baseline ECG	201	20	68.5	36	56	18	16	12	30-days mortality
García-Granja 2021 [43]	Spain	Retrospective	Hospitalized patients	ECG	517	54	68.1	44	50	18	9	9	In-hospital mortality
Harrison 2020 [51]	United States	Retrospective	Patients ≥ 50 years	ICD-10 codes	68,975	2455	65.2	52	46	24	NA	NA	NA
Kelesoglu 2020 [40]	Turkey	Retrospective	Hospitalized patients	ECG, telemetry	658	33	54	43	32	18	9	0	In-hospital mortality
Lanza 2020 [25]	Italy	Prospective	ED admission	Baseline ECG	324	20	65.9	34	52	11	14	NA	In-hospital mortality
Li 2020 [26]	China	Retrospective	Hospitalized patients	Baseline ECG	135	8	64 *	49	33	15	17	NA	NA
Linschoten 2020 [27]	Multinational	Prospective	Hospitalized patients	ECG	3011	142	67 *	37	45	23	28	NA	NA
Maeda 2020 [28]	United States	Retrospective	Hospitalized patients	ECG	181	9	64.0	44	65	34	18	13	NA
Mountantonakis 2021 [45]	United States	Retrospective	Hospitalized patients	ECG, medical notes	9564	1687	64.8	41	63	40	20	7	In-hospital mortality
Musikantow 2021 [41]	United States	Retrospective	Hospitalized patients	ICD-9/10 codes	3970	375	66 *	42	34	25	16	8	In-hospital mortality
Pardo Sanz 2020 [29]	Spain	Prospective	Hospitalized patients	ECG, ECG Holter	160	12	65.7	40	47	16	4	19	In-hospital mortality
Peltzer 2020 [30]	United States	Retrospective	Hospitalized patients	ECG, telemetry	1053	166	62.4	62	54	30	33	9	In-hospital mortality
Piroth 2020 [31]	France	Retrospective	Hospitalized patients	ICD-10 codes	89,530	11,129	65	47	33	19	16	NA	NA
Poterucha 2020 [32]	United States	Retrospective	Hospitalized patients	Baseline ECG	887	46	64.1	42	61	39	NA	NA	30-days mortality
Rav-Acha 2020 [12]	Israel	Retrospective	Hospitalized patients	ECG changes	390	20	57.5 *	45	30	20	10	7	In-hospital mortality
Russo 2020 [10]	Italy	Retrospective	ED admission	Baseline ECG	414	71	66	38	64	26	NA	17	In-hospital mortality
Sala 2020 [11]	Italy	Prospective	Hospitalized patients	Baseline ECG	132	8	65	33	45	20	0	12	NA
Santoro 2020 [33]	Italy/Germany	Prospective	Hospitalized patients	Baseline ECG	110	6	58	33	39	13	6	NA	NA
Spinoni 2021 [42]	Italy	Retrospective	Hospitalized patients	ECG	637	134	NA	NA	NA	NA	NA	NA	In-hospital mortality
Vee 2020 [34]	Malaysia	Retrospective	Hospitalized patients	NA	247	2	28 *	30	11	7	2	NA	NA
Wang 2020 [35]	China	Retrospective	Hospitalized patients	Baseline ECG	319	20	65	52	44	23	30	NA	NA
Wetterslev 2021 [44]	Denmark	Retrospective	Severe hospitalized patients	ECG, medical notes	155	52	66 *	27	44	21	100	NA	NA
Yenerçag 2020 [36]	Turkey	Prospective	Hospitalized patients w/out AF, CKD, HF	ECG changes	140	13	51.7	51	47	34	NA	0	NA
Zylla 2021 [37]	Germany	Retrospective	Hospitalized patients	ECG changes	139	11	61.1	34	43	19	39	0	In-hospital mortality

Legend: * median values; AF = atrial fibrillation; CKD = chronic kidney disease; CRIT = critical; DM = diabetes mellitus; ECG = electrocardiogram; ED = emergency department; F = females; HTN = hypertension; ICD = International Classification of Diseases; NA = not available.

**Table 2:** Univariable and multiple meta-regression * analysis for AF prevalence.

Variable	Coefficient	Standard Error	Lower 95% CI	Upper 95% CI	*p*	R^2^
*Univariable Analysis*						
**Age**	0.054	0.016	0.021	0.087	0.003	0.289
**Female sex**	0.101	1.854	−3.710	3.911	0.957	0.000
**Hypertension**	2.058	0.881	0.247	3.869	0.028	0.143
**Diabetes**	1.826	1.130	−0.501	4.153	0.119	0.117
**Geographical location**					0.067	0.191
*Europe (ref.)*						
*North America*	−0.331	0.277	−0.900	0.238	0.242	
*Asia/other*	−0.702	0.287	−1.291	−0.113	0.021	
*Multiple Analysis*					0.019	0.460
**Age**	0.041	0.018	0.002	0.079	0.038	
**Hypertension**	0.115	1.357	−2.692	2.922	0.933	
**Diabetes**	3.081	1.855	−0.756	6.917	0.110	
**Geographical location**						
*Europe (ref.)*						
*North America*	−0.676	0.344	−1.387	0.036	0.062	
*Asia/other*	−0.589	0.348	−1.308	0.131	0.104	

Legend: * Maximum likelihood; for other acronyms, please see previous tables legends.

**Table 3:** Association of clinical characteristics with AF.

**Variable**	**N° Studies**	**MD**	**95% CI**	**I^2^**
*Continuous Variables*				
**Age**	7	13.2	10.5–15.9	86%
**Variable**	**N° Studies**	**OR**	**95% CI**	**I^2^**
*Categorical Variables*				
**Female sex**	8	0.83	0.76–0.90	7%
**Hypertension**	8	2.49	2.25–2.75	0%
**Diabetes**	8	1.38	1.24–1.54	0%
**CHF**	7	4.45	3.21–6.18	58%
**CAD**	6	2.57	2.05–3.21	58%
**Critical status**	12	3.62	2.39–5.48	69%

Legend: AF = atrial fibrillation; CAD = coronary artery disease; CHF = congestive heart failure; CI = confidence interval; MD = mean difference; OR = odds ratio.

## Supplementary Materials

The following are available online at https://www.mdpi.com/article/10.3390/jcm10112490/s1: Table S1: Full Search Strategy, Table S2: Bias Assessment—NOS for prevalence of AF, Table S3: Bias Assessment—NOS for outcomes according to AF, Table S4: Sensitivity Analysis for the prevalence of AF according to different analysis methods, Table S5: Pre-specified subgroup analysis for AF prevalence, Table S6: Univariable and Multivariable Meta-Regression Analysis for All-Cause Mortality, Figure S1: PRISMA Flow-Chart of the Study, Figure S2: Leave one out analysis for prevalence of AF, Figure S3: Leave one out analysis for all-cause mortality according to AF, Figure S4: Subgroup analysis according to definition of all-cause mortality (In-hospital mortality vs. 30-days mortality), Figure S5: Publication Bias for all-cause mortality according to AF, Figure S6: Sensitivity analysis on New-Onset AF.
