# Supplementary material for: Prevalence and Impact of Atrial Fibrillation in Hospitalized Patients with COVID-19: A Systematic Review and Meta-Analysis
Source: J Clin Med. 2021 Jun 4;10(11):2490. doi: 10.3390/jcm10112490 (PMC8200114; doi:10.3390/jcm10112490)
Supplement: Supplementary file 1 [file jcm-10-02490-s001.zip › jcm-1256064-SI.pdf]

Journal of
Clinical Medicine

MDPI

Supplementary Materials

Prevalence and Impact of Atrial Fibrillation in Hospitalised patients with COVID-19: A Systematic Review and Meta-Analysis

Supplementary Table S1. – Full Search Strategy.

PubMed
#1 "severe acute respiratory syndrome coronavirus 2"[Supplementary Concept] OR "severe acute respiratory syndrome coronavirus 2"[All Fields] OR "ncov"[All Fields] OR "2019-nCoV"[All Fields] OR "COVID-19"[All Fields] OR "SARS-CoV-2"[All Fields] OR ((coronavirus[All Fields] OR "cov"[All Fields]))
#2 "sars-cov-2"[MeSH Terms] OR "sars-cov-2"[All Fields] OR "sars cov 2"[All Fields]
#3 "Covid-19"[All Fields] OR "Covid"[All Fields]
#4 #1 OR #2 OR #3
#5 ("atrial fibrillation"[MeSH Terms] OR ("atrial"[All Fields] AND "fibrillation"[All Fields])) OR "atrial fibrillation"[All Fields]
#6 #4 AND #5
**EMBASE**
#1 'coronavirus disease 2019'/exp
#2 Covid
#3 'severe acute respiratory syndrome coronavirus 2′
#4 'sars-cov-2'
#5 #1 OR #2 OR #3 OR #4
#6 ‘atrial fibrillation′
#7 #5 AND #6

Supplementary Table S2. – Bias Assessment – NOS for prevalence of AF.

Study	Selection (2)	Comparability (1)	Outcome (2)	Total (5)
Abe 2020*,**	2	0	1	3
Angeli 2020*,***	1	1	1	3
Bhatla 2020	2	1	2	5
Chen 2020*,#,***	0	1	1	2
Colon 2020	2	1	2	5
Coromilas 2021	2	1	2	5
D’Andrea 2020*	1	1	1	3
Denegri 2020*	2	1	1	4
Harrison 2020*,a	1	1	1	3
García-Granja 2021	2	1	2	5
Kelesoglu 2020	2	1	2	5
Lanza 2020**	2	0	2	4
Li 2020*	2	1	1	4
Linschoten 2020	2	1	1	4
Maeda 2020	2	1	2	5
Mountantonakis 2021	2	1	2	5
Musikantow 2021	2	1	2	5
Pardo Sanz 2020	2	1	2	5

Peltzer 2020	2	1	2	5
Piroth 2020*	2	1	1	4
Poterucha 2020*,**	2	0	1	3
Rav-Acha 2020*	2	1	1	4
Russo 2020	2	1	2	5
Sala 2020[a,b]	1	1	1	3
Santoro 2020*,[b]	1	1	1	3
Spinoni 2021	1	1	1	3
Vee 2020	2	1	0	3
Wang 2020*	2	1	1	4
Yenercag 2020*,[b]	1	1	1	2
Wetterslev 2021#,[a]	1	1	1	3
Zylla 2021*,**	2	0	1	3

Legend: *Missing/incomplete reporting of baseline charateristics; **Some patients were excluded from the main analysis due to incomplete data; ***Enrolled less than 100 patients; #Included only critical/severe patients; [a]Potential bias in the definition of AF during COVID-19; [b]Potential selection bias; NOS= Newcastle-Ottawa Scale.

Supplementary Table S3. - Bias Assessment – NOS for outcomes according to AF.

Study	Selection (4)	Comparability (2)	Outcome (3)	Total (9)
Bhatla 2020	4	1	3	8
Denegri 2020*	3	0	3	6
García-Granja 2021	4	1	3	8
Kelesoglu 2021	4	2	3	9
Lanza 2020	3	1	3	7
Mountantonakis 2021	4	2	3	9
Musikantow 2021	4	2	3	9
Pardo Sanz 2020	4	2	3	9
Peltzer 2020	4	2	3	9
Poterucha 2020	3	1	3	7
Rav-Acha 2020	4	1	3	8
Russo 2020	4	2	3	9
Spinoni 2021**	2	0	3	5
Zylla 2021	4	0	2	7

Legend: *Ascertainment of endpoints potentially biased; **Incomplete/Missing baseline charcteristics NOS= Newcastle-Ottawa Scale.

Supplementary Table S4. – Sensitivity Analysis for Pooled Prevalence of AF according to different analysis methods.

Method	Prevalence	95%CI	Tau	Tau^2	I^2
**IV, Logit Transformation**	8.3%	6.6-10.5%	0.660	0.436	99.3%
**IV, FT**	8.7%	6.7-10.9%	0.099	0.010	99.5%

Legend: FT: Freeman-Tukey Double Arcsine Transformation; IV=Inverse Variance.

Supplementary Table S5. – Pre-specified subgroup analysis for AF prevalence.

Subgroups	N° Studies	Pooled Prevalence	95% CI	I^2
		Geographical Location (p for subgroup differences=0.016)		
**North America**	9	7.5%	5.0-11.0%	99.7%
**Europe**	13	11.3%	8.2-15.4%	91.7%
**Asia/Other**	9	5.3%	3.4-8.0%	95.8%
		Study Type (p for subgroup differences=0.064)		
**Retrospective**	25	8.4%	6.3-11.2%	99.5%
**Prospective**	6	5.8%	4.4-7.6%	41.2%

	<u>Risk of Bias</u> (p for subgroup differences=0.557)			
**Low Risk**	18	8.5%	6.7-10.7%	97.0%
**High Risk**	13	7.1%	4.2-11.8%	98.4%

Legend: AF= Atrial Fibrillation; CI= Confidence Interval.

Supplementary Table S6. – Univariate and Multivariable Meta-Regression* Analysis for All-Cause Mortality.

Variable	Coefficient	Standard Error	Lower 95 CI	Upper 95 CI	P	R^2
<u>Univariate Analysis</u>						
**Age**	-0.034	0.041	-0.123	0.056	0.424	0.040
**Female Sex**	-0.254	4.190	-9.476	8.967	0.953	0.000
**Hypertension**	-1.118	1.867	-5.225	2.989	0.561	0.000
**Diabetes**	-3.552	2.152	-8.287	1.184	0.127	0.114
**Geographical Location**					0.852	0.000
Europe (ref.)	-	-	-	-	-	
North America	-0.192	0.417	-1.110	0.727	0.655	
Asia/Other	0.131	0.695	-1.399	1.662	0.854	
<u>Multivariable Analysis</u>					0.547	
**Age**	-0.027	0.058	-0.164	0.110	0.654	
**Hypertension**	4.222	4.435	-6.264	14.708	0.373	
**Diabetes**	-10.709	7.372	-28.141	6.723	0.190	0.000
**Geographical Location**						
Europe (ref.)	-	-	-	-	-	
North America	1.085	1.232	-1.828	3.999	0.408	
Asia/Other	0.786	1.532	-2.837	4.409	0.624	

Legend: *Restricted Maximum Likelihood; Knapp-Hartung method; AF= Atrial Fibrillation; CI= Confidence Interval.

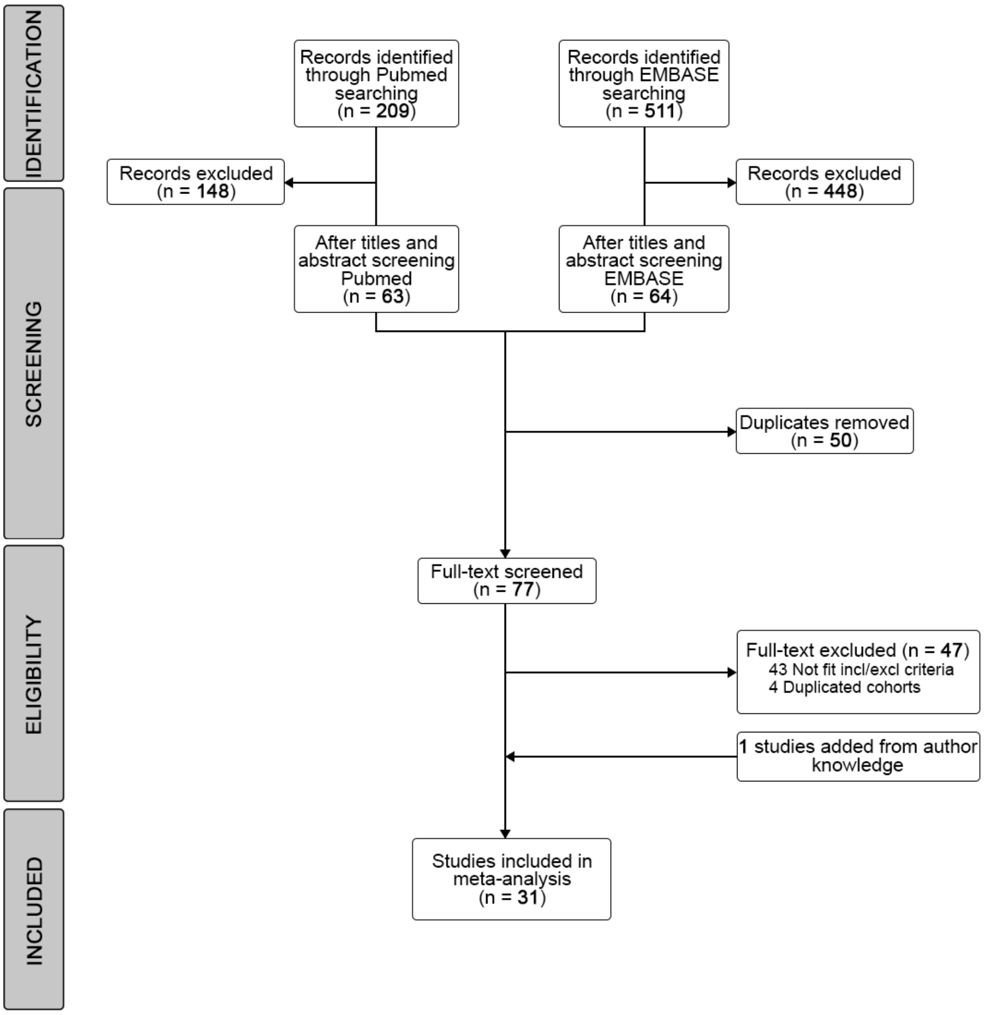

Supplementary Figure S1. – PRISMA Flow-Chart of the Study.

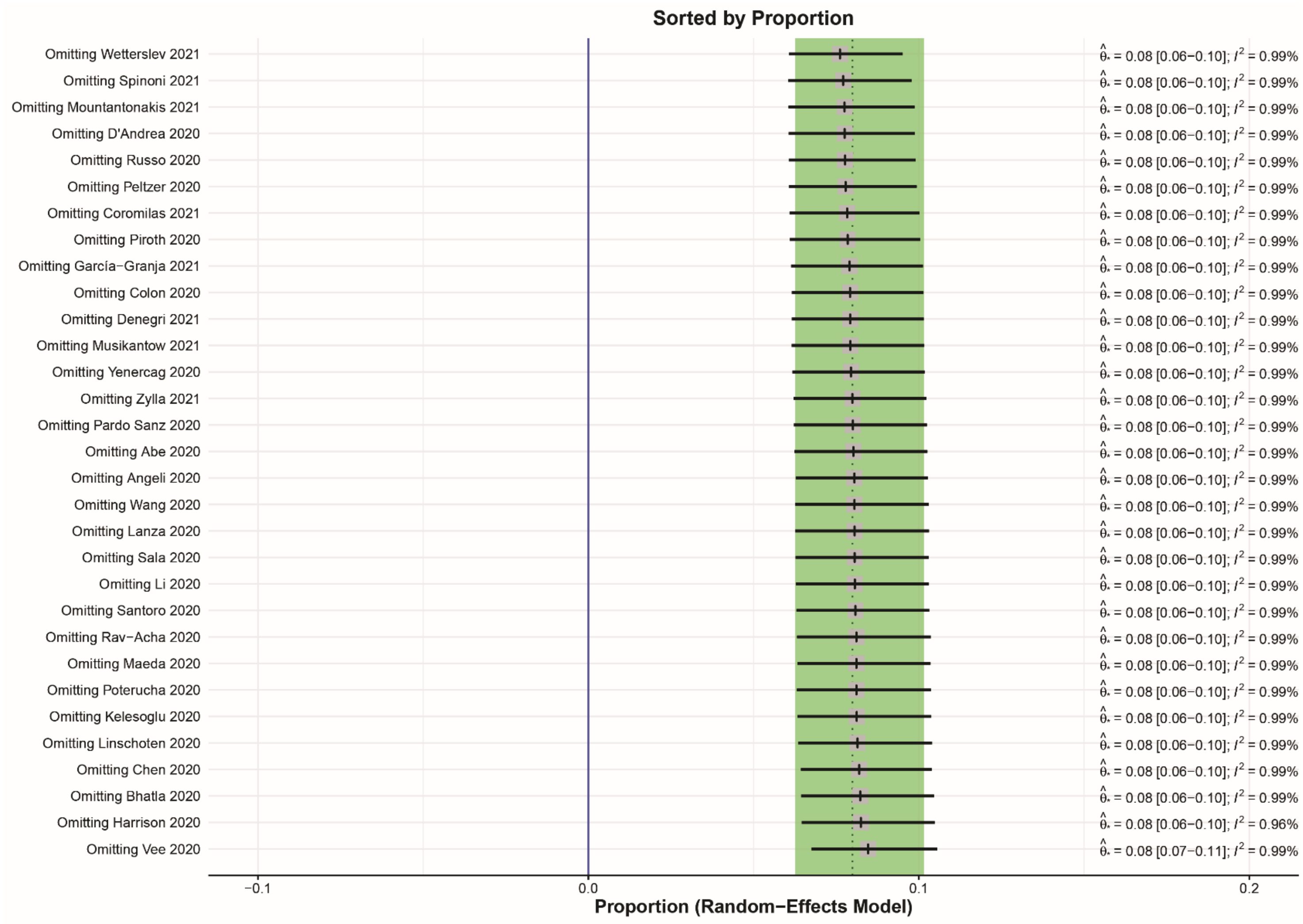

Supplementary Figure S2. – Leave one out analysis for AF Prevalence. Legend: AF= Atrial Fibrillation.

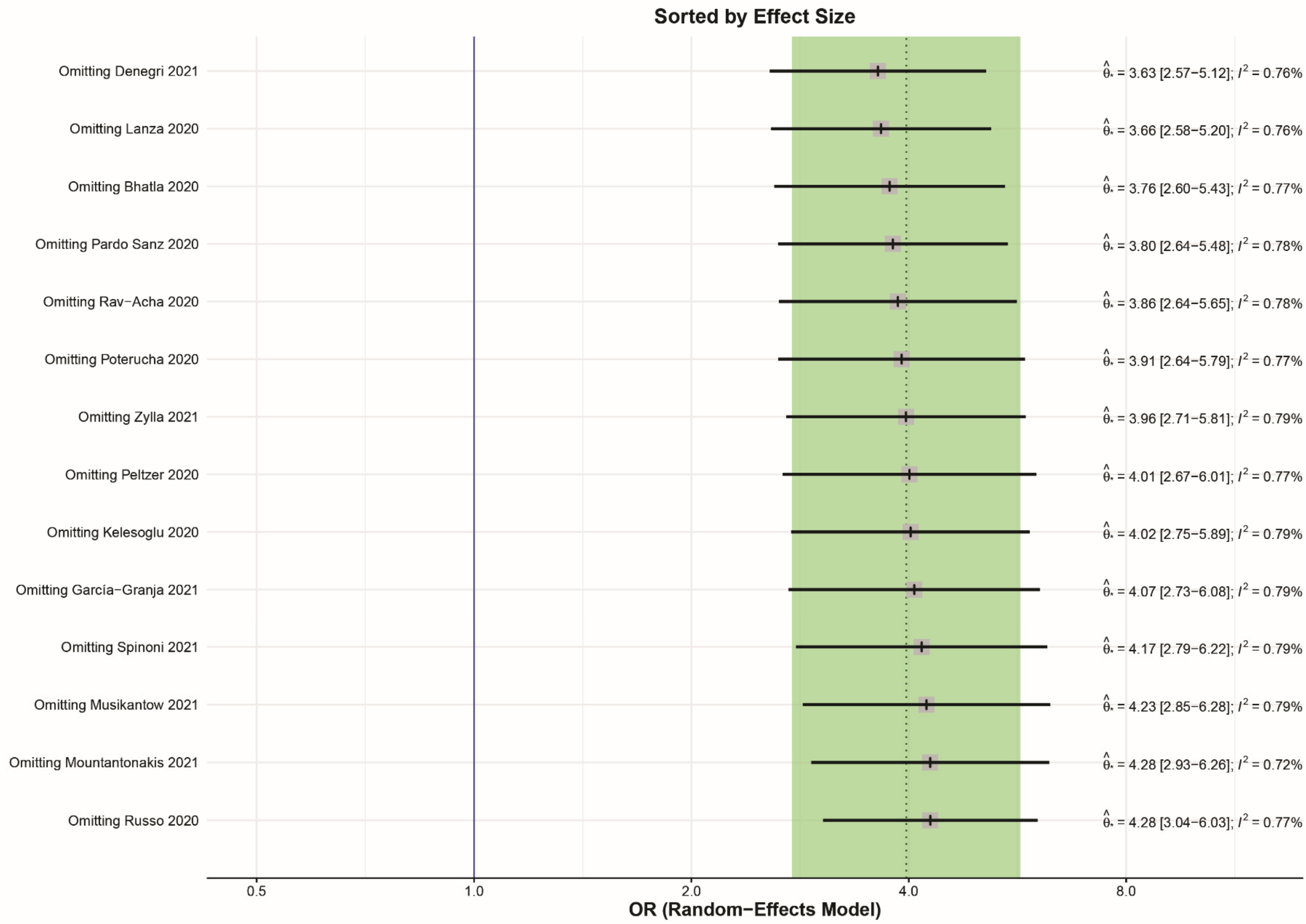

Suplementary Figure S3. – Leave one out analysis for All-Cause Death according to AF diagnosis. Legend: AF= Atrial Fibrillation; OR= Odds Ratio.

Figure S4 – Subgroup analysis according to the definition of outcome (In-hospital mortality vs. 30-days mortality)

Study or Subgroup	AF Events	AF Total	No AF Events	No AF Total	Weight	Odds Ratio MH, Random, 95% CI
**Outcome = In−hospital mortality**						
Russo 2020	21	71	86	343	8.5%	1.26 [0.71; 2.21]
Mountantonakis 2021	672	1238	460	1238	10.5%	2.01 [1.71; 2.36]
Musikantow 2021	171	375	933	3595	10.3%	2.39 [1.93; 2.97]
Spinoni 2021	59	134	111	503	9.5%	2.78 [1.86; 4.15]
Kelesoglu 2020	2	33	12	625	3.7%	3.30 [0.71; 15.37]
García−Granja 2021	27	54	103	463	8.4%	3.50 [1.96; 6.22]
Peltzer 2020	65	166	119	887	9.7%	4.15 [2.88; 5.99]
Zylla 2021	4	11	14	128	4.3%	4.65 [1.21; 17.91]
Rav−Acha 2020	6	20	23	370	5.7%	6.47 [2.27; 18.39]
Bhatla 2020	6	25	24	675	5.9%	8.57 [3.14; 23.38]
Pardo Sanz 2020	4	12	7	148	4.1%	10.07 [2.43; 41.67]
Lanza 2020	7	20	13	304	5.5%	12.05 [4.12; 35.27]
**Total (95% CI)**		**2159**		**9279**	**86.0%**	**3.52 [2.44; 5.10]**
Heterogeneity: $Tau^2 = 0.2735$; $Chi^2 = 43.68$, df = 11 ($P < 0.01$); $I^2 = 75\%$						
**Outcome = 30−days mortality**						
Poterucha 2020	27	46	181	841	8.2%	5.18 [2.82; 9.53]
Denegri 2021	14	20	28	181	5.7%	12.75 [4.52; 35.99]
**Total (95% CI)**		**66**		**1022**	**14.0%**	**7.34 [3.11; 17.34]**
Heterogeneity: $Tau^2 = 0.2168$; $Chi^2 = 2.15$, df = 1 ($P = 0.14$); $I^2 = 53\%$						
**Total (95% CI)**		**2225**		**10301**	**100.0%**	**3.97 [2.76; 5.71]**
Heterogeneity: $Tau^2 = 0.3224$; $Chi^2 = 58.59$, df = 13 ($P < 0.01$); $I^2 = 78\%$						

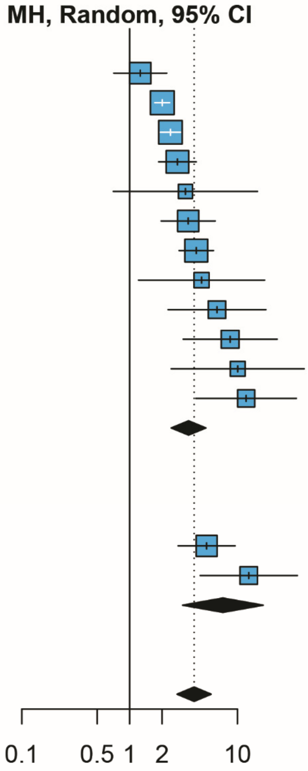

Supplementary Figure S4. – Subgroup analysis according to the definition of outcome (In-hospital mortality vs. 30-days mortality). **Legend:** AF= Atrial Fibrillation; CI= Confidence Interval; MH= Mantel-Haenszel.

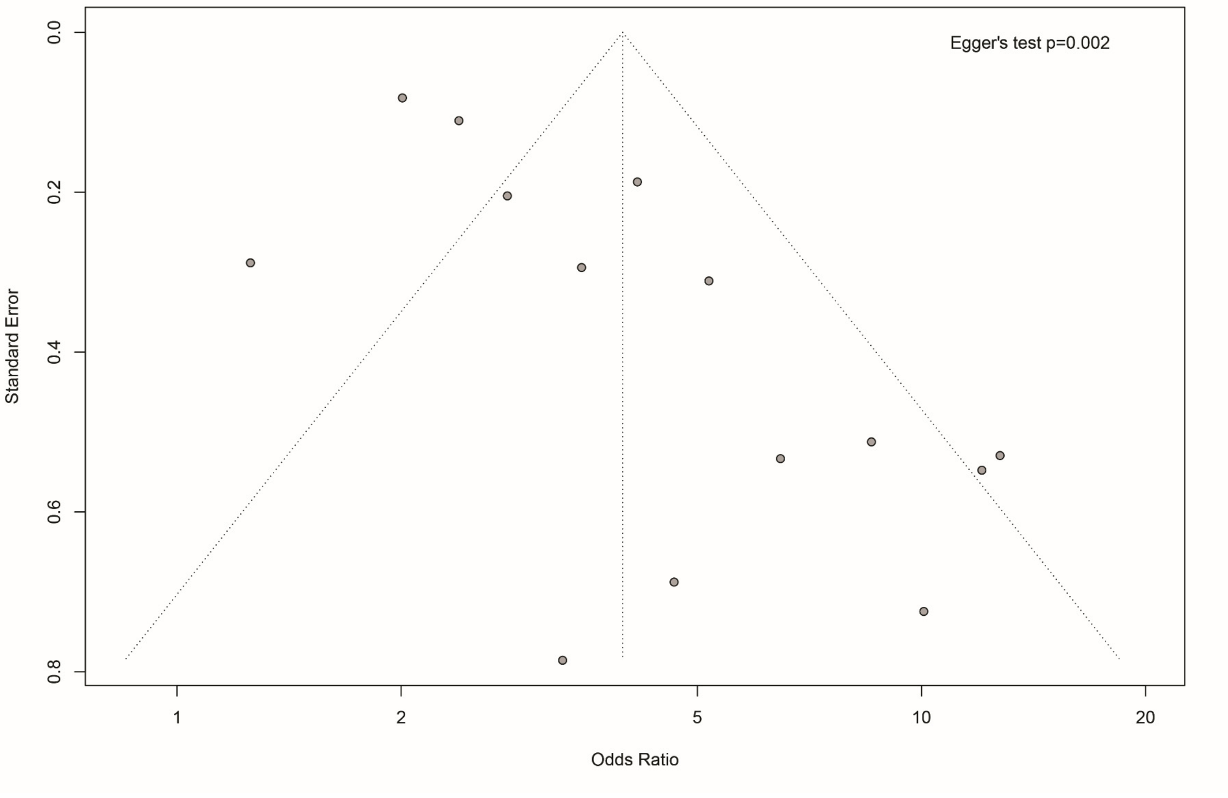

Suplementary Figure S5. – Publication Bias for all-cause death according to AF status.

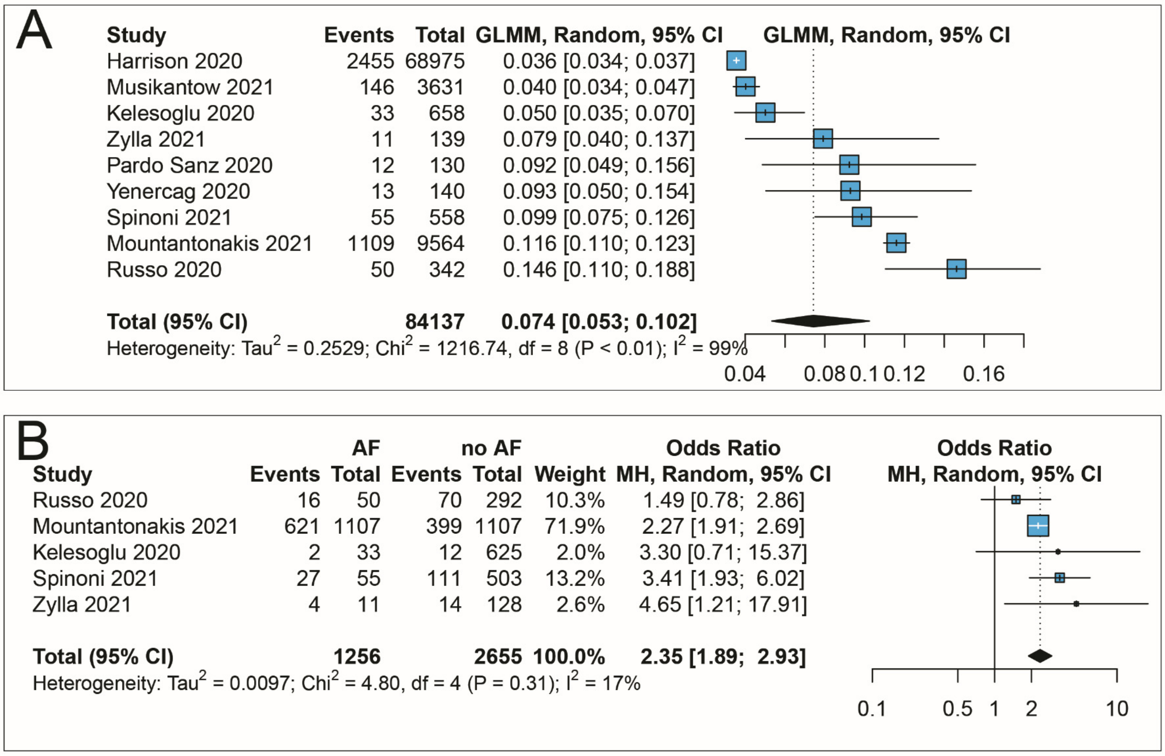

Supplementary Figure S6. – Sensitivity analysis on New-Onset AF. Legend: Panel A: Pooled prevalence of New-Onset AF; Panel B: All-cause death according to New-Onset AF status; AF= Atrial Fibrillation; CI= Confidence Interval; GLMM= General Linear Mixed Model; MH= Mantel-Haenszel.
